# Human Activity Recognition Through Augmented WiFi CSI Signals by Lightweight Attention-GRU

**DOI:** 10.3390/s25051547

**Published:** 2025-03-02

**Authors:** Hari Kang, Donghyun Kim, Kar-Ann Toh

**Affiliations:** School of Electrical and Electronic Engineering, Yonsei University, Seoul 03722, Republic of Korea; hariver1220@yonsei.ac.kr (H.K.); kimd@yonsei.ac.kr (D.K.)

**Keywords:** human activity recognition, time-series signals, WiFi CSI, self-attention, GRU, pruning, data augmentation

## Abstract

In this study, we investigate human activity recognition (HAR) using WiFi channel state information (CSI) signals, employing a single-layer gated recurrent unit (GRU) with an attention module. To overcome the limitations of existing state-of-the-art (SOTA) models, which, despite their good performance, have substantial model sizes, we propose a lightweight model that incorporates data augmentation and pruning techniques. Our primary goal is to maintain high performance while significantly reducing model complexity. The proposed method demonstrates promising results across four different datasets, in particular achieving an accuracy of about 98.92%, outperforming an SOTA model on the ARIL dataset while reducing the model size from 252.10 M to 0.0578 M parameters. Additionally, our method achieves a reduction in computational cost from 18.06 GFLOPs to 0.01 GFLOPs for the same dataset, making it highly suitable for practical HAR applications.

## 1. Introduction

Human activity recognition (HAR) is a technology designed to automatically detect and categorize various human activities. It utilizes data collected from sources such as wearable sensors, smartphones, and ambient signals. HAR plays a crucial role in various fields, including healthcare, where it monitors elderly or chronic patients [1,2,3,4], and smart homes, where it optimizes home systems through activity recognition [5,6,7,8,9]. In industrial environments, HAR improves safety by monitoring worker or driver movements [10,11,12], and supports urban planning and smart city development through public space activity analysis [13,14,15].

Several modalities have been used to recognize human activities, including vision-based approaches [16,17,18] and sensor-based methods [19,20,21,22,23,24]. However, these modalities have inherent limitations. Vision-based HAR may have a limited field of view and requires sophisticated image processing algorithms. In addition, the use of the visible spectrum can be intrusive and raise privacy concerns. Similarly, smartphone-based HAR, which utilizes built-in sensors such as accelerometers and gyroscopes, may not accurately represent whole-body activity as it is limited to the movements of the device itself.

HAR based on WiFi CSI [25,26] addresses these shortcomings. Unlike camera-based systems, CSI-based HAR does not require a direct line of sight and is less intrusive, making it particularly suitable for privacy-sensitive environments such as homes or healthcare facilities. CSI has been proven to be effective in classifying fine-grained movements, such as hand gestures [27], by capturing minute variations in wireless signals. Additionally, utilizing existing WiFi infrastructure to collect CSI data is cost-effective and easier to deploy than setting up specialized sensors or camera networks. Most modern WiFi routers can be configured to provide CSI data, which, when combined with machine learning algorithms, can significantly improve the accuracy of activity recognition over time.

Previous works such as [28,29] have relied on heavy and deep models to achieve performance improvements. Although effective, these models entail increased computational complexity and longer inference times. Studies such as [30,31] have leveraged GRU and attention modules to capture features of time-series data. To enhance inference efficiency and improve the generalization of GRU with attention on limited datasets, we incorporate pruning and data augmentation techniques. Our main contribution is thus the development of a lightweight system with the following components. (This article is a revised and expanded version of a paper entitled ‘Human Activity Recognition based on the GRU with Augmented Wi-Fi CSI Signals’, which was presented at IEEE TENCON, 1–4 December 2024.)

- A comprehensive system that utilizes a GRU-based architecture as the backbone for time-series feature representation. Following the extraction of GRU features, a self-attention mechanism is applied to focus on the most relevant activity information. The model is subsequently pruned to reduce weight redundancy, thereby significantly improving efficiency while maintaining high accuracy. An ablation study is conducted to analyze the impact of pruning on accuracy, demonstrating that our method effectively preserves performance despite a substantial reduction in model complexity.
- Several augmentation techniques are applied to address the small sample size of the available WiFi CSI dataset. The augmentation, in combination with pruning, helps prevent overfitting and enhances model generalization, while also reducing computational costs. Our experiments further assess how different augmentation strategies contribute to maintaining high accuracy in a resource-constrained environment.
- Extensive experimental evidence across multiple datasets is provided to support our evaluation, validating the model’s robustness and applicability in various scenarios.

Our paper is organized as follows: Section 2 reviews the related literature, highlighting existing methods and their limitations. Section 3 provides the necessary preliminaries, introducing the basic self-attention and GRU mechanisms, which form the foundation of the proposed model, and the introducing training methods such as pruning and fine-tuning. Section 4 introduces a lightweight model aimed at overcoming the shortcomings of SOTA approaches by integrating a single-layer GRU with an attention mechanism. This section also elaborates on the training process, including pruning and fine-tuning strategies. Section 5 presents experimental evidence demonstrating the effectiveness of the proposed method, featuring hyperparameter justification, ablation studies, and comparisons with SOTA methods. Finally, we conclude with a comprehensive summary of the contributions and findings in Section 6.

## 2. Related Works

This section provides a brief review of existing works in WiFi CSI-based human activity recognition, categorized into four main types: Hand-crafted, CNN, RNN, and Transformer methods, as summarized in Table 1.

### 2.1. Hand-Crafted Methods

Hand-crafted methods involve manually designing features based on domain knowledge; these are then used in classification algorithms. For example, Zou et al. [32] proposed multiple kernel representation learning (MKRL) to extract and fuse CSI features across domains, classifying four activities: sitting, standing, walking, and running. Guo et al. [33] applied principal component analysis (PCA) and discrete wavelet transform (DWT) for noise reduction, followed by classifiers like Naïve Bayes (NB), Random Forest (RF), Decision Trees (DTs), K-Nearest Neighbors (KNN), and Support Vector Machines (SVMs) to categorize 16 activities.

Yan et al. [34] introduced the AACA algorithm, which dynamically adjusts decision thresholds to balance performance and scalability, recognizing activities such as pushing, kicking, and running. In Huang et al. [35], Huang et al. [35] developed PhaseAnti, a WiFi-based HAR system that extracts CSI features using PCA-based subcarrier fusion and DWT, followed by kNN-DTW classification of nine motions. In Guo et al. [36], six machine learning algorithms, including SVM, Multi-layer Perceptron (MLP), Decision Tree, Random Forest, Logistic Regression, and KNN, were implemented to recognize eight types of daily activity.

In summary, hand-crafted methods offer low computational complexity, their reliance on predefined features limits their adaptability to dynamic environments. In contrast, our approach eliminates manual feature engineering by leveraging automated feature extraction from raw CSI data, enhancing flexibility and scalability across diverse scenarios.

### 2.2. CNN-Based Methods

CNN-based methods utilize convolutional layers to automatically extract and learn important features from WiFi CSI data. These approaches can be categorized into 3D-CNN, 2D-CNN, and 1D-CNN methods. In the 3D-CNN approach, WiFi CSI signals are treated as 2D spatial inputs plus a temporal frame. For example, Zhang et al. [37] proposed WiGesID, which applies 3D convolutions to segment WiFi CSI data and classify five gestures, such as push-and-pull and draw zigzag.

In the 2D-CNN approach, WiFi CSI signals are treated as 2D inputs, enabling the network to process spatial relationships within the data. For instance, Ma et al. [27] used a 9-layer CNN to classify 276 sign gestures. In the study of Moshiri et al. [38], the CSI data were converted into RGB images for each activity, using them as inputs for a CNN to classify seven daily activities such as walking, running, sitting down, and bending. Yadav et al. [28] introduced CSITime, combining convolutional layers with self-attention to recognize six hand movements. In addition, Zhang et al. [43] proposed a CSI imaging module that converts CSI data into images using five methods [59,60,61] for converting time-series data into images, Gramian angular sum field (GASF), Gramian angular difference field (GADF), Markov transition field (MTF), recurrence plot (RP), and short-term Fourier transform (STFT), before CNN-based classification. Chen et al. [46] introduced Wisor-DL, integrating gated temporal convolutional networks and a dendrite network for daily activity recognition in diverse environments. In Zhao et al. [47], a multi-channel CNN with GAM is used to extract spatial features, while a hybrid model combines CNN for local feature extraction, LSTM, and self-attention for recognizing six movement types, including stretching and body turning. Finally, an approach based on the 1D CNN processes WiFi CSI signals as univariate time series, focusing on temporal features. Memmesheimer et al. [29] applied signal reduction and EfficientNet to extract features for six activity classifications using the ARIL dataset. Chen et al. [46] used 1D convolutions for temporal feature extraction, while Li et al. [49] introduced WCNN, a 1D CNN-based model that captures both local and time-correlated features, classifying activities across multiple datasets.

In summary, methods in the 3D-CNN approach capture spatial features along with temporal relationships, while 2D-CNN methods transform CSI time-series data into a 2D spatial format for feature extraction. However, these 3D and 2D data transformations introduce significant computational overhead, often requiring additional modules to capture temporal dynamics effectively. In contrast, 1D-CNN-based methods offer greater efficiency for processing univariate signals, making them more scalable across diverse datasets and activities. Nonetheless, 1D CNNs may fail to capture spatial correlations among signals, which limits their ability to model complex interactions.

### 2.3. RNN-Based Methods

Next, RNN-based methods utilize sequential data by maintaining hidden states that capture temporal dependencies between inputs, allowing the network to model time-dependent patterns. RNN-based methods are commonly categorized into LSTMs and GRUs, which are both designed to effectively handle sequential dependencies in time-series data.

In LSTM methods, the ability to capture long-term dependencies makes LSTMs well suited for modeling complex temporal patterns. Chen et al. [25] enhanced a Bidirectional LSTM (BLSTM) with an attention mechanism to capture past and future CSI features, improving classification across seven activities. Ding et al. [26] applied wavelet-based denoising and extracted time-frequency features using FFT and STFT before LSTM-based classification of six activities. Zhang et al. [50] employed PCA, a band-pass filter, and STFT to generate spectrograms, training a Dense-LSTM model for ten activity classes. Shang et al. [51] combined an LSTM with a CNN to jointly extract spatial and temporal features, evaluating two datasets with five daily activity classes.

In GRU methods, a simplified architecture that uses fewer parameters than in LSTMs, while capturing temporal dependencies, is deployed. Shalaby et al. [52] introduced a CNN-GRU hybrid, where convolutional layers extract features before a GRU processes sequential data, improving activity classification across six movements such as lie down, fall, and stand up. Kang et al. [53] developed a GRU network that integrates both past and current inputs within each cell to enhance the capture of temporal relationships. To address the challenge of limited data, they employed three data augmentation techniques: adding Gaussian noise, shifting, and CutMix. These methods expanded the dataset by approximately 20 times, allowing the model to learn more robust feature representations. The model was evaluated on ARIL [39], SignFi [27], and HAR [54], achieving SOTA performance.

Unlike existing SOTA methods, our approach takes a more data-driven and computationally efficient direction. Specifically, we design a model-friendly architecture by carefully structuring a single-layer GRU combined with an attention module, ensuring that both the temporal axis and hidden dimension information of the time-series data are effectively captured. This design allows for a more data-adaptive representation while reducing model complexity. Furthermore, we introduce a model optimization strategy that includes structured pruning and fine-tuning, significantly reducing computational cost while maintaining high classification performance. To further enhance the model’s generalization ability, we incorporate multiple data augmentation techniques, including time-series shifting, MixUp, and adding Gaussian noise, leading to improved robustness against variations in WiFi CSI data. Compared to previous works, our method not only achieves SOTA results on multiple datasets but also offers a lightweight yet expressive solution, making it more suitable for real-world on-device applications.

### 2.4. Transformer Methods

Lastly, we explore Transformer methods. These methods can model long-range dependencies without the constraints of sequential processing, enabling Transformers to capture complex patterns in time-series data. For example, Yang et al. [55] proposed two modified Transformer architectures to enhance WiFi-based human gesture recognition: the United Spatiotemporal Transformer (UST) and the Separated Spatiotemporal Transformer (SST). The SST uses two distinct encoders to intuitively capture spatial and temporal features, while the UST employs a single encoder to efficiently extract three-dimensional spatiotemporal features due to its optimized structure. This model was tested on Widar3.0 [56], which comprises 22 gestures. Other examples include Bian et al. [57], who implemented a Swin Transformer-based autoencoder–decoder for CSI feature compression, tested on a custom 22-class dataset. Luo et al. [58] explored five Vision Transformer architectures (ViT, SimpleViT, DeepViT, Swin Transformer, CaiT) for WiFi CSI-based HAR. Their models were evaluated on UT-HAR (lay down, pick up, fall, sit down, run, walk, stand up) and NTU-Fi (running, boxing, cleaning floor, walking, falling down, circling arms).

Comparing with the above methods, our implementation addresses several key limitations of traditional RNN methods. While RNNs excel at capturing temporal dependencies, our implemented GRU-attention model leverages both an attention mechanism and a pruning strategy with data augmentation to effectively mitigate the inherent limitations of RNN variants. Specifically, the attention mechanism selectively emphasizes the most informative temporal segments, reducing the reliance on uniformly processing all inputs, while the pruning strategy eliminates redundant computations, thereby lowering the overall computational overhead. Additionally, this attention-driven focus enhances the interpretability of the model’s outputs and ensures that the network maintains robust performance even when confronted with long and complex sequences. Unlike pure RNN-based approaches, the data augmentation techniques we employ improve robustness and generalization, which are critical in noisy, real-world environments. Thus, by integrating GRU architectures with attention, pruning, and data augmentation techniques, our model not only preserves the temporal modeling strengths of RNNs but also achieves a favorable balance among performance, efficiency, and robustness compared with standard RNN-based methods.

## 3. Preliminaries

In this section, we present some essential preliminaries that are necessary to understand before discussing our developed system. We provide an overview of key techniques such as self-attention, GRU, pruning, and fine-tuning. These methods enhance model performance and efficiency by focusing on various aspects of capturing sequential data while reducing computational costs.

### 3.1. Self-Attention

Self-attention [62] is a mechanism used in neural networks that enables the model to assess and evaluate the relevance of different tokens within an input sequence. The self-attention mechanism is implemented using three learnable weight matrices, **$W_{Q}$**, **$W_{K}$**, and **$W_{V}$**, which transform the input into query, key, and value matrices, respectively. The attention scores are calculated as the dot product of the query and key matrices, followed by a softmax operation to obtain the attention weights. These weights are then used to compute a weighted sum of the values, resulting in the output of the self-attention layer. Mathematically, the self-attention mechanism can be represented as

$$\mathrm{Attention}\left(Q,K,V\right)=\mathrm{Softmax}\left(\frac{QK^{T}}{\sqrt{d_{k}}}\right)V$$

where **Q**, **K**, and **V** are the query, key, and value matrices, and $d_{k}$ is the dimension of the keys.

### 3.2. GRU

A gated recurrent unit (GRU) [63] simplifies the architecture of long short-term memory (LSTM) [64], leading to reduced memory requirements and computational costs. With fewer parameters, GRUs are particularly well suited for smaller datasets and incorporate an effective gating mechanism consisting of update and reset gates:

- Update gate ($z_{t}$): The update gate determines how much of the past information should be retained and passed forward to future time steps, enabling selective retention or forgetting of information. It is defined as
  $$z_{t}=\sigma \left(W_{z}\cdot \left[h_{t-1},x_{t}\right]\right),$$
  where $x_{t}$ is the input at time step *t*, $h_{t-1}$ is the hidden state from the previous time step, $W_{z}$ is the weight matrix for the update gate, and σ is the sigmoid activation function that constrains the output to the range [0,1].
- Reset gate ($r_{t}$): The reset gate decides how much of the past information should be forgotten, allowing the model to discard irrelevant details. It is defined as
  $$r_{t}=\sigma \left(W_{r}\cdot \left[h_{t-1},x_{t}\right]\right)$$
  where $W_{r}$ is the weight matrix for the reset gate, $h_{t-1}$ is the previous hidden state, and σ is again the sigmoid activation function.

By linking up these gates, the mathematical formulation of a GRU can be written as follows:

$${\tilde{h}}_{t}=\tanh\left(W_{h}\cdot \left[r_{t}⊙h_{t-1},x_{t}\right]\right)$$

$$h_{t}=\left(1-z_{t}\right)⊙h_{t-1}+z_{t}⊙{\tilde{h}}_{t}$$

where $h_{t}$ is the hidden state at time *t*, representing the memory of the network at that time step, and ${\tilde{h}}_{t}$ is the candidate hidden state, which is a potential update for the hidden state. The symbol ⊙ represents element-wise multiplication.

### 3.3. Network Pruning and Fine-Tuning

Pruning is a technique employed to reduce the size of neural networks by eliminating less important weights or neurons. This approach aims to enhance the computational efficiency of the model, decrease memory usage, and potentially improve the generalization ability of the model. The existing strategies for pruning include weight pruning, neuron pruning, and structured pruning. Weight pruning involves setting individual weights to zero based on their magnitude. Neuron pruning removes entire neurons or filters. Structured pruning, however, systematically eliminates groups of parameters, such as entire channels or layers. Our proposed system adopts weight pruning.

Fine-tuning leverages the knowledge learned from the initial training on a large dataset and adapts the knowledge to a specific domain with a smaller dataset. Essentially, the performance of the model is enhanced on the new task by transferring the learned features and representations from the original task, thus reducing the amount of data and computational resources needed for training.

## 4. Proposed System

In this section, we present a system for recognizing human activities using WiFi CSI data. The system consists of a lightweight Attention-GRU with three training and regulating components: data augmentation, model pruning, and fine-tuning. The goal is to develop an efficient and accurate system for HAR by leveraging these components. We call the system Attention-GRU for convenience.

### 4.1. System Overview

**Figure 1:**
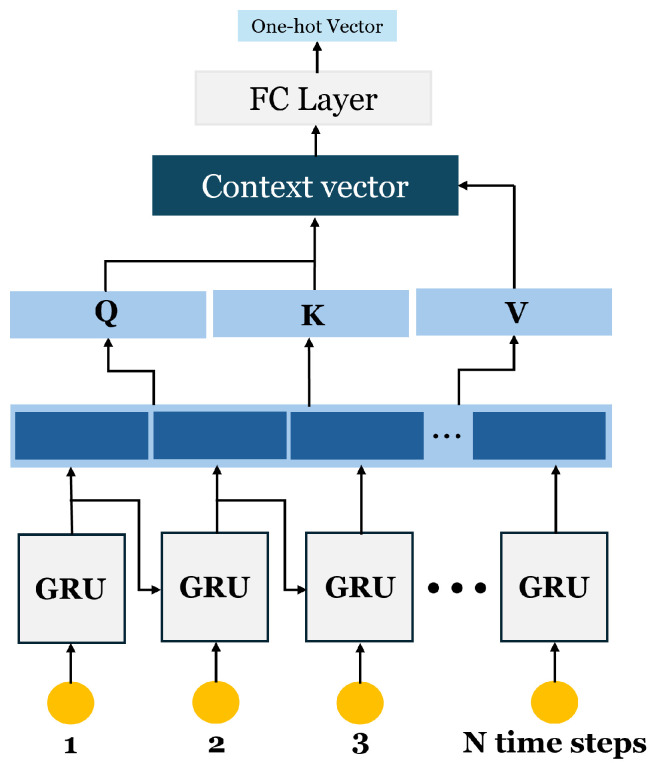
Attention-GRU network: The original time-series data are processed through the GRU, which extracts representative features. These features are then passed to the attention module for final classification by a fully connected layer.

The proposed Attention-GRU consists of a single GRU layer with 128 units, followed by a self-attention module with a hidden dimension of 32. The architecture is depicted in Figure 1. Essentially, the GRU layer processes the raw time-series data to convert them into a more expressive representation. Each data sample is a three-dimensional input of shape (data samples × sequence length × channels), where *channels* corresponds to multiple subcarrier measurements recorded at each time step of the sequence. Each time step is sequentially fed into the GRU cell, enabling the model to capture temporal dependencies across the entire sequence. The resulting transformed data are then passed through a self-attention module to emphasize the most relevant features while suppressing less important ones. The network parameters are trained considering the limited data size. Three data augmentation techniques are employed to improve generalization.

### 4.2. Attention-GRU

The GRU is selected as the backbone of our system. Unlike the LSTM, which maintains separate cell states and hidden states, the GRU combines these into a unified representation. This design reduces the number of parameters and computational complexity, resulting in faster training and inference, which is especially advantageous in online monitoring applications. In comparison with a Bi-LSTM, which learns bidirectional dependencies by processing data in both the forward and backward directions, a GRU offers a lighter and more computationally efficient alternative. While a Bi-LSTM can capture more complex temporal dependencies, it comes at the cost of increased computational requirements and memory usage, which may not be practical in resource-constrained environments such as wearable devices. A single-layer GRU, on the other hand, effectively captures the sequential characteristics of HAR data while ensuring a lightweight model design. Considering these factors, a GRU strikes a balance between performance and efficiency, making it an appropriate choice as the backbone for our HAR system.

Essentially, the GRU first enhances the original time-series input—which includes multiple channels per time step—by effectively capturing and representing the explanatory power of these features. This process ensures that each time step’s multidimensional input is mapped into a more expressive and informative sequence, accounting for the nuances across different channels. The transformed sequence is then passed through a self-attention module to reinforce temporal correlations and highlight the most relevant features. In this module, the key, query, and value vectors are derived from the GRU output itself. The query vector interacts with the key vector to compute attention scores, which are subsequently used to weight the value vector. By selectively emphasizing critical segments of the temporal sequence, the attention mechanism further refines the model’s focus, enabling more accurate recognition of human activities.

### 4.3. Network Training, Pruning, and Fine-Tuning

Pre-training: The first step in our method involved pre-training the Attention-GRU architecture using data augmented with the Gaussian noise, shifting, and MixUp techniques, as detailed in Section 4.4. This pre-training served as the initial foundation model.

Pruning: Once pre-training was completed, pruning was applied to reduce the number of model weights by setting a selected proportion, *k*, of the weights—those whose absolute values were below a certain threshold, *s*—to zero. This approach systematically reduced model complexity while retaining the most significant weights. By removing only the less significant weights (those below the threshold *s*), the pruning process ensured that the model’s ability to learn important features was not overly compromised. The optimal ratio was determined experimentally by adjusting the threshold for pruning the weights based on validation accuracy.

**Figure 2:**
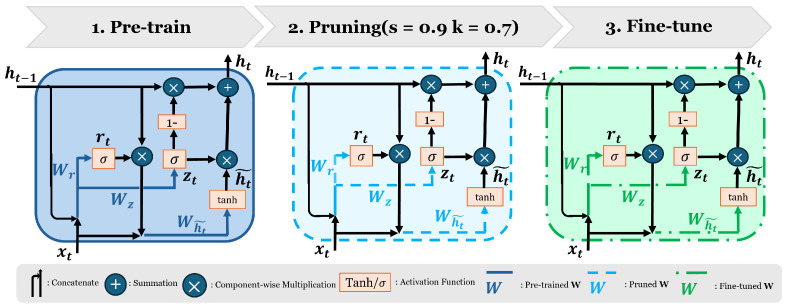
Training and pruning pipeline: Augmented data (Gaussian noise and shifting) were used to pre-train the Attention-GRU model. The pre-trained weights (**W**) were then pruned and subsequently fine-tuned for network customization. This figure illustrates the GRU structure prior to the attention module, with weight colors representing pre-trained (dark blue), pruned (light blue), and fine-tuned (green) weights.

Fine-tuning: After pruning, fine-tuning was necessary to overcome the inevitable performance degradation that can occur when pruning alone is applied. To achieve this, we fine-tuned the pruned model using a lower learning rate than the one used during the pre-training phase. The flow of pre-training, pruning, and fine-tuning is depicted in Figure 2.

### 4.4. Data Augmentation

In view of the limited availability of WiFi CSI data containing human activities, we adopt data augmentation techniques to expand the dataset. Specifically, we employ three techniques: adding Gaussian noise, shifting, and incorporating MixUp [42].

#### 4.4.1. Adding Gaussian Noise

Adding Gaussian noise to data during augmentation can improve a model’s generalization ability by simulating real-world variability, making the model more robust to noise and minor variations. This helps the model focus on underlying data patterns rather than memorizing specific details, ultimately enhancing its performance on unseen data. In the proposed system, Gaussian noise with a mean of 0 and a variance $\sigma^{2}$ of 0.0001 is added to the original signal to create a noise-perturbed version for subsequent processing. Before augmentation, our dataset is normalized using a standard scaler, transforming each channel’s data to have a mean of 0 and a variance of 1. Given this normalized range, a variance $\sigma^{2}$ of 0.0001 provides subtle regularization, preserving the data’s intrinsic patterns while preventing overfitting.

Consider a time-series training matrix $X\in R^{m\times (T\times d)}$, where *m* refers to the number of training samples, *T* refers to the sequence of data, and *d* represents the number of channels, the noise perturbed matrix $\tilde{X}$ is obtained as follows:

$$\tilde{X}=X+X⊙N,$$

where $N\in R^{m\times (T\times d)}$ stands for the Gaussian noise matrix and the Hadamard product (⊙) is a matrix operation that performs element-wise multiplication between two matrices with identical dimensions.

The augmented dataset is generated by adding noise to the original dataset three times, resulting in a fourfold expansion. Correspondingly, the training target label matrix, denoted as $Y\in R^{m\times c}$, where *c* is the number of classes, is stacked to match the augmentation. The final augmented matrices, $X_{\mathrm{aug}}\in R^{4m\times (T\times d)}$ and $Y_{\mathrm{aug}}\in R^{4m\times c}$, are thus four times the size of the original dataset:

$$\begin{array}{cc} X_{\mathrm{aug}}=\left[\begin{array}{c} X\\ \tilde{X}\\ \tilde{X}\\ \tilde{X} \end{array}\right] & Y_{\mathrm{aug}}=\left[\begin{array}{c} Y\\ Y\\ Y\\ Y \end{array}\right] \end{array}.$$

**Figure 3:**
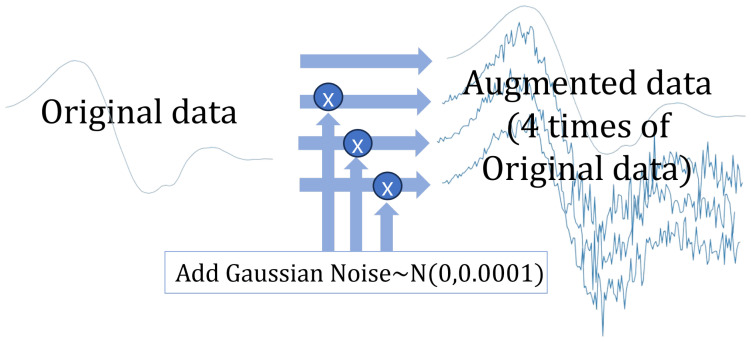
Adding Gaussian noise: This figure illustrates the augmentation method using a single channel of a time-series data sample. The top sequence represents the original signal. By applying the noise-adding augmentation technique, three augmented data samples are generated for the same channel. This process is applied to all channels.

Figure 3 illustrates the process of augmentation via adding Gaussian noise.

#### 4.4.2. Data Shifting

In time-series data related to human activities, variations in the start and end of motions can significantly affect recognition accuracy. To improve the model’s generalization, temporal shifts are applied as a data augmentation technique. By shifting the sequences forward and backward along the time axis, the model is exposed to variations in temporal alignment while retaining the core structure of the data.

Suppose $X_{i}\in R^{d\times T}$ represents a single sample of data comprising column vectors $\left[x_{1},x_{2},\ldots ,x_{T}\right]$, where each $x_{i}\in R^{d}$. Here, *T* represents the sequence length, and *d* denotes the channel dimension. Applying temporal shifts of one to three time steps forward results in the following augmented sequences:

$${\tilde{X}}_{\mathrm{shift}\text{\_}1\mathrm{step}}=\left[x_{2},x_{3},\ldots ,x_{T-2},x_{T-1},x_{T},x_{1}\right]$$

$${\tilde{X}}_{\mathrm{shift}\text{\_}2\mathrm{steps}}=\left[x_{3},x_{4},\ldots ,x_{T-1},x_{T},x_{1},x_{2}\right]$$

$${\tilde{X}}_{\mathrm{shift}\text{\_}3\mathrm{steps}}=\left[x_{4},x_{5},\ldots ,x_{T},x_{1},x_{2},x_{3}\right]$$

**Figure 4:**
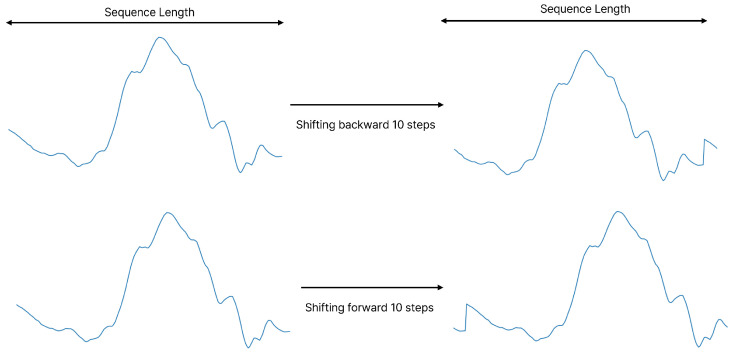
Data shifting: This figure illustrates a forward and backward shift of 10 time steps. Including such shifted signals during training helps the model recognize shifted signals in the test set.

In these sequences, each time step is shifted forward by one, two, or three positions. Figure 4 illustrates the data augmentation process through temporal shifting. This figure reveals signal discontinuities at the junctures where the front and end segments meet. These discontinuities can be considered as an intentional addition of noise to the data, which forms another key step in our data augmentation process. Notably, this approach preserves the essential features that represent the progression of the action.

#### 4.4.3. MixUp

**Figure 5:**
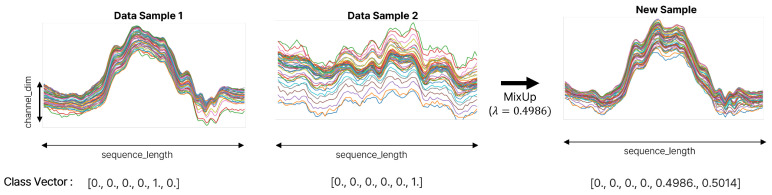
MixUp: New samples are generated by linearly combining data from two different classes based on a mixing coefficient λ, drawn from a beta distribution Beta(α,α). The left and middle plots represent pure samples (respectively, at λ=1 and at λ=0), while the right plot shows a mixed sample with category proportions λ=0.4986 and 1−λ=0.5014.

As illustrated in Figure 5, the MixUp process creates augmented inputs and labels as smooth interpolations between two original samples. By blending features and labels randomly and proportionally, MixUp encourages the model to learn more robust representations, enhancing its generalization ability and smoothing decision boundaries.

## 5. Experiments

In this section, we present the experiments conducted to evaluate the proposed system. This includes descriptions of the datasets used, the experimental setups, and the process for determining the best hyperparameters of the pruned model. We also analyze how each data augmentation technique affects the model’s recognition performance. Finally, we compare our model’s results with other leading methods to benchmark its effectiveness in WiFi CSI-based human activity recognition.

### 5.1. Databases

We employed four public datasets for our experimentation: the ARIL dataset [39], the StanWiFi dataset [40], the Sign-Fi dataset [27], and the Nexmon HAR dataset [54]. The links to these datasets are listed in Table 2.

The ARIL dataset includes six hand movements—hand up, hand down, hand left, hand right, hand circle, and hand cross—performed across 16 locations, each repeated 15 times, resulting in 1440 samples (1116 training and 324 test samples), with 192 time steps and 52 channels.

The StanWiFi dataset comprises 4478 samples, each containing 500 time steps with amplitudes from 30 channels across three antennas. It includes six activities: fall, run, sit down, stand up, lay down, and walk. The dataset was collected from six subjects in an indoor environment, with each action repeated 20 times. A WiFi router acted as a transmitter, and a computer with an NIC 5300 network card and three antennas served as the receiver. The transmitter and receiver were positioned 3 m apart in a line-of-sight (LOS) setup, and the sampling frequency was 1000 Hz.

The Sign-Fi dataset consists of wireless signal characteristics of sign gestures through CSI measurements. It comprises 8280 instances representing 276 sign gestures, collected in both lab (5520 samples) and home environments (2760 samples). These gestures were gathered from five users, and raw CSI data were pre-processed to remove noise and recover meaningful variations over subcarriers and time.

The Nexmon HAR dataset consists of 1201 samples, including 960 training and 241 test samples, each containing 500 time-series arrays with 256 channels. The experimental setup included a transmitter–receiver pair where the receiver was in monitoring mode, capturing CSI data using Nexmon-patched firmware. Activities were recorded in an indoor environment, with UDP packets generating controlled network traffic. The dataset includes human activities such as empty, lying, sit, sit down, stand, stand up, walk, and fall.

### 5.2. Experimental Settings

We conducted three experiments to evaluate the proposed system. Experiment I aimed to determine the best hyperparameter settings for feature extraction using the ARIL dataset. Experiment II conducted an ablation study to assess the impact of individual mechanisms on system performance using the ARIL dataset. Experiment III compared the proposed system with SOTA methods across four datasets. The experiments are summarized in Table 3. As the ARIL dataset is already split into training and test sets, we further partitioned the training set into training and validation subsets for tuning parameters. The model that achieved the highest validation score during training was saved and later evaluated on the test set.

For datasets other than ARIL, which do not provide predefined splits, we ensured consistency by manually dividing them into training, validation, and test subsets. The training and validation sets were used exclusively for model parameter tuning, while five-fold cross-validation was employed to assess the methodology, with separate test sets used to evaluate performance. This method ensures a robust evaluation by averaging performance across multiple folds, thereby minimizing the impact of potential bias in the data split. Throughout this process, we also ensured that the test set was neither augmented nor involved in any training-related procedures. This strict separation prevents data leakage and ensures that the reported performance accurately reflects the model’s generalization capabilities.

To optimize model performance, we set the initial learning rate to 0.0001 and employed a cosine annealing learning rate scheduler, where the maximum number of iterations was aligned with the total number of epochs. The Adam optimization algorithm was chosen for its adaptive learning capabilities, and early stopping was implemented to halt training if no further improvement was observed before reaching 100 epochs. Experiments were conducted using an RTX 3060 GPU. This efficiency highlights the computational advantages of our approach compared with existing studies. The combination of an optimized deep learning model and an effective training strategy contributed to improved performance while maintaining computational feasibility. Table 4 provides a detailed overview of the hyperparameters used in our experiments.

### 5.3. Experiment I: Finding the Best Hyperparameters

#### 5.3.1. Determining the Best Hidden Dimensions of Attention-GRU

The ARIL dataset is utilized to determine the optimal hidden dimensions of the Attention-GRU model. A single-layer GRU with an attention module is adopted for feature extraction. The extracted features are then linearly transformed to output a one-hot vector. The model undergoes pre-training, pruning, and fine-tuning to identify the best hidden dimensions.

Table 5, Table 6 and Table 7 present the comprehensive results of experiments conducted with various combinations of hyperparameters, while Table 8 shows the key findings of recognition accuracy across different GRU and attention dimensions, focusing only on meaningful combinations from the overall experimental results. From this table of key findings, we observe that a GRU dimension of 256 combined with an attention dimension of 128 achieves the highest accuracy of 99.28%. Conversely, a GRU dimension of 128 combined with an attention dimension of 32 demonstrates the best balance between performance and computational efficiency, achieving competitive accuracy while significantly reducing computational overhead.

#### 5.3.2. Network Pruning

The subsequent stage is to prune the network in order to achieve a lightweight model. A weight threshold *s* is used as the masking parameter by comparing the absolute values of the weights to it. Weights with absolute values less than the threshold are set to zero, thus reducing the model size. A predefined ratio *k* represents the proportion of weights to be kept in the model. This ratio is crucial to prevent performance degradation after pruning.

Fine-tuning is then conducted to ensure the pruned model maintains its performance. Table 9 shows the results for different pruning ratios and threshold settings. The pruning ratios and thresholds were selected from k,s∈{0.1,…,0.9} based on the best performance. These results indicate that setting k=0.7 and s=0.9 achieves a balance between performance and sparsity.

#### 5.3.3. Data Augmentation by Adding Gaussian Noise

To determine the best setting for adding Gaussian noise, we observe the performance of the model under different noise variances $\sigma^{2}\in \left\{0.00001,0.0001,0.001,0.01\right\}$. The impact of these hyperparameters on the overall effectiveness of the augmentation method is shown in Table 10. Based on these results, the variance $\sigma^{2}=0.0001$ achieved the highest accuracy of 94.61%, demonstrating that the level of noise injection should neither be too high nor too low for good generalization.

#### 5.3.4. Data Augmentation with MixUp

To determine the best setting for MixUp, we observe the performance under different reflection ratios r∈{0.3,0.5,0.8,1} and mixing strengths α∈{0.5,1,1.5,2}. The impact of these hyperparameters on the overall effectiveness of the augmentation method is shown in Table 11. From this table, we observe variations in recognition accuracy at different combinations of *r* and α settings. In particular, when α=1 and r=0.7, the model achieves the best performance, with an accuracy of 97.84%. This setting suggests a balancing between the strength of mixing and the reflection ratio to produce the highest model accuracy.

#### 5.3.5. Data Augmentation by Temporal Shifting

To determine the best shifting time steps, we observe the performance under different step sizes n∈{1,2,3,4,5,10,20}. The shifting augmentation was implemented pairwise, involving both shiftings in the forward (positive shift) and backward (negative shift) directions. As observed from Table 12, the accuracy improves consistently with larger step sizes, indicating that broader shifts help enhance model generalization. Specifically, the accuracy increases from 88.49% at step size 1 (containing a positive shift step and a negative shift step) to a peak of 98.92% at step size 10 (containing 10 positive shift steps and 10 negative shift steps). This trend suggests that expanding the range of shifting enables the model to better capture variations in the data, thereby improving its robustness to diverse input patterns. These results underscore the importance of selecting an optimal step size for shifting during data augmentation.

### 5.4. Experiment II: Ablation Study of Each Mechanism

#### 5.4.1. Attention-GRU with Pruning

To evaluate the effectiveness of each module modification, we performed an ablation study, as shown in Table 13. First, we compared the GRU model with and without attention before data augmentation. The GRU-only model focuses on updating the hidden state to effectively capture information at each step of the sequence. As indicated in Table 13, the GRU-only model achieves an accuracy of 68.92%. However, adding attention allows the model to capture global relationships within the sequence, leading to a performance improvement. Specifically, the Attention-GRU model’s accuracy increases to 70.86%, demonstrating that incorporating attention mechanisms enhances the model’s ability to understand the overall context in the sequence.

Next, we address the increase in parameters caused by adding attention. To compensate for the additional parameters introduced by the attention mechanism, we apply pruning to the Attention-GRU model. As shown in Table 13, pruning effectively reduces the total number of parameters from 82.1K to 57.9K, a reduction of approximately 29.5%. Although there is a slight decrease in accuracy to 69.42%, the trade-off between model size and performance is well balanced. Regarding the increase in training time despite the reduced number of parameters in the pruned Attention-GRU model, it is important to note that pruning alone does not inherently improve performance. Therefore, a fine-tuning step was employed after pruning to enhance the model’s accuracy. This fine-tuning process requires additional training time, which accounts for the observed increase. The pruning and fine-tuning workflow successfully decreases the number of parameters while mitigating the decrease in accuracy, making the model more efficient without significant performance loss.

#### 5.4.2. Data Augmentation with Tuned Hyperparameters

The upper and lower blocks of Table 13 show the model without and with data augmentation, respectively. Comparing the results between the model without data augmentation and variants utilizing various data augmentation techniques, the difference is evident. Without any augmentation, the baseline Attention-GRU model achieves an accuracy of 69.42%. When the augmentation is applied, significant performance gains are observed for all the augmentation techniques. When all three augmentation techniques—Gaussian noise, MixUp, and shifting—are applied together, the model achieves the highest accuracy of 98.92%, demonstrating the effectiveness of combining these methods. However, the removal of each augmentation technique reveals their individual contributions to performance. Removing Gaussian noise causes a slight drop in accuracy to 97.12%, indicating that Gaussian noise contributes to performance improvement but is not as impactful as the other techniques. In contrast, removing MixUp leads to a more substantial decrease, with accuracy falling to 89.93%, suggesting that MixUp plays an important role in improving generalization. Finally, the removal of shifting results in the most dramatic drop, reducing accuracy to 83.81%, which underscores shifting as the most critical augmentation technique for achieving high performance.

To observe the impact of shifting on model performance, we experimented with different step sizes by applying backward and forward shifts of 1, 2, 3, 4, 5, 10, and 20, as shown in Table 12. Among these, the step size of 10 was identified as achieving the highest accuracy. The observed changes in accuracy across the various step sizes were relatively small and consistent, indicating that the model is robust to such variations. Furthermore, the controlled and incremental adjustments suggest that the augmentation process does not inadvertently introduce data leakage, thereby maintaining the integrity of the training data while effectively enhancing the model’s generalization capability. This comparison demonstrates the substantial impact of augmentation techniques on improving model performance. The use of augmentation not only enhances accuracy but also ensures that the model remains efficient with a reduced number of parameters, as evidenced by the pruning performance.

### 5.5. Experiment III: Comparison with SOTA

In this experiment, we applied all augmentation techniques to the final model and compared its performance with SOTA methods. As shown in Table 14, the proposed model achieved the highest accuracy across all four datasets: 98.92% for ARIL [39], 99.33% for StanFi [40], 99.32% for Sign-Fi [27], and 100% for HAR [54], outperforming or matching the SOTA models. Moreover, the proposed model demonstrated superior efficiency by significantly reducing the number of GFLOPs and achieving the minimum number of parameters across all four datasets: 0.01 GFLOPs and 0.0578 M parameters for ARIL [39], 0.0083 GFLOPs and 0.0680 M parameters for StanFi [40], 0.005 GFLOPs and 0.2818 M parameters for Sign-Fi [27], and 0.1495 GFLOPs and 0.1571M parameters for HAR [54].

Existing methodologies for the ARIL [39] and Sign-Fi [27] datasets have utilized approaches such as multiple convolutional layers, residual connections, self-attention mechanisms, and EfficientNet-based architectures, as shown in the methodologies of CSITime [28] and Gimme Signal [29]. In contrast, our approach applies a single-layer GRU combined with an attention module, achieving model lightweighting while maintaining superior performance. In terms of the StanFi [40] dataset, STC-NLSTMNet achieved state-of-the-art performance by combining depthwise separable convolution (DS-Conv) for spatial feature extraction, a feature attention module (FAM) to emphasize significant spatial features, and nested LSTM (NLSTM) for capturing long-term temporal dependencies in CSI signals. In contrast, our approach achieves similar performance with fewer parameters, demonstrating its efficiency. For the HAR [54] dataset, LSTM and SVM achieved state-of-the-art performance. In contrast, we propose a more lightweight model that not only maintains similar performance but also enhances robustness by incorporating various augmentation techniques.

In addition to the overall performance improvements, the proposed model also demonstrated superior results in experiments under different conditions for the Sign-Fi dataset, as shown in Table 15. Specifically, the proposed model outperformed the baseline (CSITime [28]) across all subsettings, including “Lab”, “Home”, “Lab + Home”, and “Lab-5”.

These results highlight the superiority of the proposed strategy, which combines all augmentation techniques with pruning. The proposed model not only achieves the highest accuracy across all datasets but also significantly reduces computational complexity and memory requirements, making it a highly efficient and accurate solution for the studied tasks.

### 5.6. Summary of Results and Discussion

In this section, we summarize the experiments conducted, highlight the significant results obtained, and discuss the key insights observed.

#### 5.6.1. Summary of Results

Table 16 summarizes the results from the experiments, highlighting the efficacy of the proposed methods. In experiment I, the optimal GRU and attention dimensions (128 and 32, respectively) achieved an accuracy of 98.56%. Pruning further improved this accuracy to 98.93%, while MixUp augmentation validated the importance of data augmentation, achieving 97.84% accuracy. Experiment II showed that adding attention increased the number of parameters by 10% but boosted accuracy by 1.94%, while pruning reduced the number of parameters by 25% with a 0.50% accuracy gain. Data augmentation methods, particularly shifting, consistently improved performance. Finally, experiment III highlighted significant computational efficiency, achieving 0.72% higher accuracy with 1000× fewer GFLOPs compared to ARIL [39] and outperforming or matching other SOTA models like StanWiFi [40], Sign-Fi [27], and HAR [54] with reduced parameters.

#### 5.6.2. Discussion

Our model demonstrated remarkable performance in classifying six distinct gestures, achieving an accuracy of 98.56% on the ARIL dataset. Notably, it attained perfect accuracy (100%) for gestures such as “hand up”, “hand down”, “hand left”, and “hand cross“ on the HAR dataset. This exceptional performance can be attributed to the GRU’s ability to effectively retain critical sequential information and the attention mechanism’s strength in isolating task-relevant features, thereby reducing the impact of noise and irrelevant motion patterns. Together, these components work synergistically to deliver robust classification, even for gestures with subtle variations.

**Figure 6:**
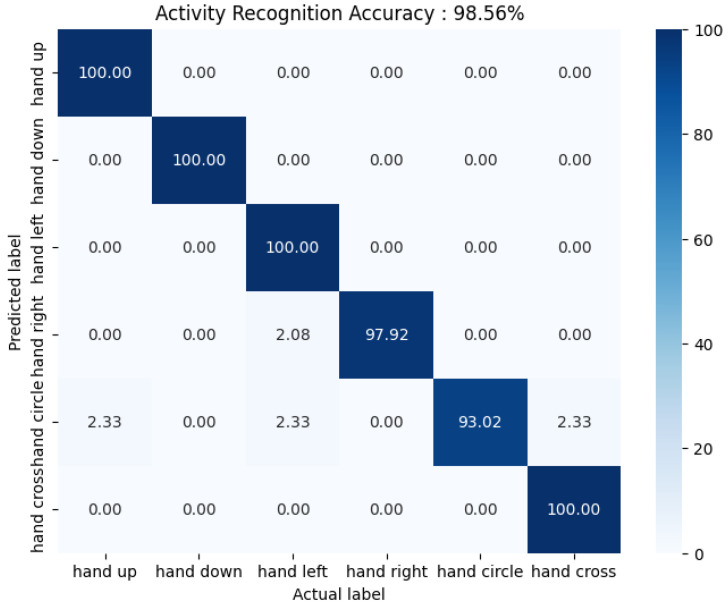
Confusion matrix of six gestures from the ARIL dataset [39].

However, an analysis of the misclassified cases for the ARIL dataset, as illustrated in Figure 6, revealed occasional errors. For instance, the model sometimes mislabeled the “hand left” gesture as “hand right” and the “hand circle” gesture as “hand up”, “hand left”, or “hand cross”. These misclassifications likely stem from the challenges associated with dynamic gestures like “hand circle”, where the starting point and motion trajectory significantly influence the model’s interpretation. For example, a circular motion that begins with an upward or lateral movement might be mistakenly identified as “hand up” or “hand left”. Additionally, the structural similarity between “hand circle” and “hand cross”—especially when gestures are performed with slight variations in precision or speed—can result in overlapping feature representations.

These observations highlight both the strengths and limitations of our approach. While our model effectively captures and classifies most gestures, it also underscores the need for more advanced spatiotemporal modeling to address subtle distinctions in gesture dynamics. Future work could focus on integrating higher-order temporal dependencies and developing precision-aware feature extraction techniques to further enhance accuracy and robustness in gesture recognition systems, particularly for real-world applications.

## 6. Conclusions

In this study, we developed a human activity recognition system using WiFi CSI signals, leveraging an Attention-GRU model. To address model complexity, we incorporated data augmentation and pruning techniques. Our experiments demonstrated that optimizing the hyperparameters for data augmentation and applying pruning effectively balanced prediction performance and computational efficiency. The optimal configuration, with a GRU dimension of 256 and an attention dimension of 128, provided the best prediction performance. Additionally, the lightweight model, which was 29.5% smaller in network size, also performed exceptionally well.

While the current system has achieved a balance between accuracy and model complexity, several directions for future research can be explored to further enhance the model’s capabilities. These include the integration of higher-order temporal dependencies to better capture complex patterns in high-dimensional time-series data. A preliminary study in [53] shows that the inclusion of additional temporal input provides possible performance improvement. Additionally, expanding the system’s robustness to handle varying levels of noise and interference through advanced augmentation techniques will be beneficial. Moreover, real-world deployment poses practical concerns, including achieving online inference and scalability across diverse hardware platforms. Reducing inference latency while maintaining accuracy is a critical challenge. Efficient model compression techniques, such as quantization and knowledge distillation, can be explored to enable deployment on resource-constrained devices without compromising performance. Furthermore, we acknowledge the importance of evaluating robustness against noise, improving scalability for larger datasets, and conducting online inference assessments in future work. These aspects will be systematically studied to ensure a more comprehensive evaluation of our approach. Future work will focus on implementing and validating these strategies to further enhance the system’s real-world applicability.

## Figures and Tables

**Table 1:** Summary of recent literature on HAR.

Method	Paper	Techniques	Dataset and Activities	Data Augmentation
Hand-crafted	Zou et al., 2017 [32]	MKRL and Auto-HSRL	4 activities	None
	Guo et al., 2019 [33]	PCA, DWT, NB, RF, DT, KNN, and SVM cassifiers	16 activities	None
	Yan et al., 2020 [34]	AACA	10 activities	None
	Huang et al., 2023 [35]	PhaseAnti, PCA, and KNN-DTW classifiers	10 activities	None
	Guo et al., 2024 [36]	SVM, MLP, DT, RF, Logistic Regression, and KNN	8 activities	Increasing the number of IMUs
CNN	Zhang et al., 2022 [37]	WiGesID(3D CNN)	Categories not in the train set	None
	Ma et al., 2018 [27]	9-layer CNN	SignFi [27]	None
	Moshiri et al., 2021 [38]	2D CNN	7 activities	None
	Yadav et al., 2022 [28]	Kernel extraction and attention module	ARIL [39], SignFi [27], StanFi [40]	CutMix [41], MixUp [42]
	Zhang et al., 2023 [43]	3-layer CNN with GASF, GADF, MTF, RP, and STFT	Wiar [33], SAR [44], Widar3.0 [45], and self-test dataset	none
	Chen et al., 2024 [46]	Dilated Conv with residual connection and Hadamard product	6 types of activity in office and lab	None
	Zhao et al., 2025 [47]	Hybrid model CNN with LSTM	6 types of activity	Data interpolation
	Memmesheimer et al., 2020 [29]	EfficientNet [48]	ARIL [39], TU RGB+D 120, and UTD-MHAD	Interpolating, sampling, scaling, filtering, adding noise
	Li et al., 2023 [49]	Wide-time-domain CNN	5 datasets with 13 to 25 activities	None
RNN	Chen et al., 2018 [25]	Attention-based ABLSTM	6 common daily activities	None
	Ding et al., 2019 [26]	HARNN, which is an LSTM with features using FFT and STFT	6 types of activity in home	None
	Zhang et al., 2021 [50]	Dense-LSTM with PCA and SIFT	10 activities	Gaussian noise, time stretch, spectrum shift, spectrum scale, frequency filter
	Shang et al., 2021 [51]	LSTM-CNN network	Static and dynamic movements	None
	Shalaby et al., 2022 [52]	CNN-GRU	6 activities	None
	Kang et al., 2024 [53]	Input-modified GRU	ARIL [39], SignFi [27], HAR [54]	Adding Gaussian noise, shifting, CutMix [41]
Transformer	Yang et al., 2021 [55]	United and Separated Spatiotemporal Transformers	Widar3.0 [56]	None
	Bian, 2024 [57]	Swin Transformer-based autoencoder–decoder	21 different classes set up in a meeting room	CutMix [41]
	Luo et al., 2024 [58]	Vision Transformer	UT-HAR, NTU-Fi HAR	None

**Table 2:** Datasets and their source links.

Dataset	Source Link
**ARIL**	https://github.com/geekfeiw/ARIL (accessed on 25 February 2025)
**StanWiFi**	https://www.researchgate.net/figure/Summary-of-the-StanWiFi-dataset_tbl2_366717201 (accessed on 25 February 2025)
**Sign-Fi**	https://yongsen.github.io/SignFi/ (accessed on 25 February 2025)
**Nexmon HAR**	https://www.semanticscholar.org/paper/Human-Activity-Recognition-Using-CSI-Information-Sch%C3%A4fer-Barrsiwal/9ddb0cf17a3ac4e9d73bd7df525ff66ab2af73d1 (accessed on 25 February 2025)

**Table 3:** Experimental settings.

Brief Descriptions of the Experiments	Databases
**Experiment I: Finding the best hyperparameter settings** (a) Determine the GRU hidden dimension and the attention module hidden dimension (b) Determine the pruning ratio *k* and threshold, *s* (c) Determine the Gaussian noise variance, $\sigma^{2}$ (d) Determine the shifting steps, *n* (e) Determine the mixing strength, $\alpha^{2}$ and epoch ratios for reflection of MixUp, *r*	ARIL [39]
**Experiment II: Ablation study of each mechanism** (a) Evaluate Attention-GRU with pruning (b) Assess data augmentation	ARIL [39]
**Experiment III: Comparison of the proposed method with SOTA**	ARIL [39] StanFi [40] Sign-Fi [27] HAR [54]

**Table 4:** Model hyperparameters.

Hyperparameter	Value
Model	1 GRU (hid dim = 128) and Attention (hid dim = 32)
Batch size	128 (after augmentation 512)
Training epochs	100 (50 in HAR dataset [54])
Adam β1	0.9
Adam β2	0.999
Learning rate	1 × 10^−3^
Scheduler	CosineAnnealingLR (T max = 100)
Loss function	Cross-Entropy Loss

**Table 5:** HAR accuracy (%) at different GRU and Attention dimensions.

GRU Dimension	Attention Dimension				
	32	64	128	256	512
32	87.77	90.29	89.57	90.29	88.13
64	95.68	94.60	93.53	96.04	95.32
128	98.56	97.48	97.48	98.20	98.56
256	98.56	98.92	99.28	98.56	98.92
512	98.56	98.56	98.56	98.56	98.56

**Table 6:** Total number of parameters (in thousands) for different GRU and Attention dimensions.

GRU Dimension	Attention Dimension				
	32	64	128	256	512
32	11.7	15.0	21.8	35.4	62.5
64	29.1	35.6	45.5	74.4	126.1
128	82.5	95.1	120.3	170.8	271.6
256	263.0	287.9	337.7	437.3	636.4
512	918.9	968.3	1067.3	1265.3	1660.9

**Table 7:** Total GFLOPs over various GRU and Attention dimensions.

GRU Dimension	Attention Dimension				
	32	64	128	256	512
32	0.00159	0.00159	0.00160	0.00161	0.00164
64	0.00436	0.00436	0.00438	0.00440	0.00445
128	0.01343	0.01344	0.01347	0.01352	0.01362
256	0.04574	0.04576	0.04581	0.04591	0.04595
512	0.04611	0.16697	0.16702	0.16732	0.16771

**Table 8:** Selected significant values from the comprehensive dimension study.

Model		Accuracy (%)	Total FLOPs (G)	Total Parameters (K)
GRU dim	Attention dim			
**128**	**32**	**98.56**	**0.0134**	**82.5**
	64	97.84	0.0134	95.1
	128	98.56	0.0135	120.3
**256**	32	98.56	0.0457	263.0
	64	98.20	0.0458	287.9
	**128**	**99.28**	**0.0458**	**337.7**
512	32	98.56	0.0461	918.9
	64	98.56	0.1670	968.3
	128	98.56	0.1670	1067.3

**Table 9:** Results of the comprehensive pruning study.

Model		Accuracy (%)	Total Parameters (K)
*k*: Prune Ratio	*s*: Threshold		
**0.7**	0.5	98.92	60.6
	0.8	98.56	57.8
	**0.9**	**98.92**	**57.8**
0.8	0.5	98.56	65.9
	0.8	98.92	65.9
	0.9	98.56	65.9

**Table 10:** Impact of Gaussian noise variance on model accuracy.

Variance $\sigma^{2}$	Accuracy (%)
0.01	92.81
0.001	93.53
**0.0001**	**94.61**
0.00001	94.24

**Table 11:** Recognition accuracy (%) at different settings of *r* and α.

	Reflection Ratio *r*	0	0.3	0.5	0.7	1
α						
**0.5**		80.22	91.73	96.40	97.12	96.40
**1.0**		82.73	96.04	96.04	**97.84**	**97.84**
**1.5**		83.09	94.60	96.76	96.76	97.84
**2.0**		82.01	95.68	95.68	96.76	97.48

**Table 12:** Performance comparison with different shifting step sizes: The positive and negative numbers in brackets indicate that both positive and negative shift augmentations were incorporated simultaneously.

**Step Sizes *n***	(−1,1)	(−2,2)	(−3,3)	(−4,4)	(−5,5)	**(** −10,10 **)**	(−20,20)
**Accuracy (%)**	88.49	91.73	94.60	94.25	97.48	**98.92**	98.20

**Table 13:** Ablation study.

Model Description	Accuracy (%)	Training Times	Total Parameters (K)
**Without Data Augmentation**			
GRU (Baseline)	68.92	1 m 48 s	69.7
Attention-GRU (Baseline)	70.86	2 m 22 s	82.1
Pruned Attention-GRU	69.42	2 m 22 s + 14 s	57.9
**With Data Augmentation**			
Shifting + MixUp	97.12	48 m 46 s + 4 m 55s	57.8
Gaussian Noise + Shifting	89.93	36 m 1 s + 3 m 35 s	57.8
Gaussian Noise + MixUp	83.81	48 m 39 s + 4 m 56 s	57.8

**Table 14:** Comparison of model performance with SOTA models across different datasets.

Dataset	Model	Accuracy (%)	GFLOPs	Total Parameters (M)
ARIL [39]	CSITime [28]	98.20	18.06	252.10
	Gimme signals [29]	94.91	1.02	25.13
	Our system	98.92	0.01	0.0578
StanFi [40]	STC-NLSTMNet [40]	99.88	0.044	0.0871
	Our system	99.33	0.0083	0.0680
Sign-Fi (lab) [27]	CSITime [28]	99.22	18.06	252.10
	Our system	99.32	0.05	0.2818
HAR [54]	LSTM [54]	98.95	3.72	0.2482
	GRU with past input [53]	100	0.245	0.2469
	Our system	100	0.180	0.1660

**Table 15:** Results of the Sign-Fi dataset under different conditions.

Model	Accuracy (%)			
	Lab	Home	Lab + Home	Lab-5
CSITime [28]	99.22	97.39	96.23	88.83
Ours	99.32	99.64	97.52	89.60

**Table 16:** Summary of experiments and key results.

Details for Each Experiment	Configuration/Observation
Best model dimension settings	GRU dim: 128, Attention dim: 32 with 98.56% accuracy
Optimal pruning ratio and threshold	k=0.7, s=0.9 with 98.93% accuracy
Best settings for MixUp	α=1.0, k=0.7 with 97.84% accuracy
Attention-GRU	+10% in total parameters and +1.94% accuracy
Attention-GRU with pruning	−25% reduction in total parameters and +0.50% accuracy
Data augmentation	Shifting > MixUp > Gaussian Noise and +20% accuracy
Comparison with ARIL	1000× fewer GFLOPs and +0.72% accuracy
Comparison with StanFi	20× fewer GFLOPs and 99.33% accuracy
Comparison with Sign-Fi	500× fewer GFLOPs and +0.1% accuracy
Comparison with HAR	100% accuracy

## Data Availability

All datasets used in the paper are publicly available. The implemented codes are available in https://github.com/harikang/prunedAttentionGRU, accessed on 25 February 2025.
